# Dilatation of the Stomach

**Published:** 1902-08-09

**Authors:** 


### DILATATION OF THE STOMACH.

A discussion on the Causes, Diagnosis, and Principles of Treatment of Dilatation of the Stomach was opened by Professor Clifford Allbutt, who devoted his remarks more particularly to the atonic and non-obstructive dilatations. In regard to etiology, he attributed a good deal to the mechanical relations of the organ, more especially in regard to the portion of the organ affected. As to the question whether dilatation was caused by gastritis or *vice versâ*, one must remember that the presence of gastritis is often assumed on very slender grounds, even our post-mortem knowledge of this condition leaving much to be desired. Relaxation of the stomach due primarily to muscular failure and appearing first in the pouch and the fundus may, he said, depend upon toxic causes or mere atony. Such gastric distension advancing to dilatation not uncommonly retards convalescence from such diseases as typhoid or rheumatic fever. Much the same condition may also follow slow digestion with fermentation, anæmia, or mere inanition, the muscular failure in such cases being primary, while in other cases associated with dyspepsia the dilatation may be a secondary consequence of spasm of the pylorus. Again, in seeking for causes of dilatation we must not overlook the nervous system, for not only may continuously acting depressing causes set up atony of the stomach, but sudden and grave mental shock may be quickly followed by atonic dilatation of the organ. Perhaps among the nervous causes one may place those by no means uncommon cases in which atonic dilatation occurs as a consequence of excessive athletic exertion or mental stress, especially in young people of delicate constitution. Finally, dilatation of the stomach is far from being rare in little children and infants, often coming on acutely but being readily relieved by emesis or the use of the tube.

The recognition of atonic dilatation is not always an entirely simple matter, the symptoms being often indefinite and misleading to an observer who is not forewarned. There may be little or no loss of appetite or dyspepsia—indeed, in an early stage food often relieves a sense of sinking which may be present—while vertigo, sleeplessness, heaviness, lassitude, fatigue and mental depression may divert attention to the nervous system. Flatulence, however, is rarely absent, and the bowels are generally constipated (occasionally lienteric), but the tongue may be clean and vomiting may be slight or absent. Then the condition is associated with many nervous symptoms. In extreme cases the pulse falls in volume and tension, the hands become livid, the face thin, drawn, and sallow.

To arrive at a diagnosis physical examination must be made at various periods of digestion, and also at times when the stomach ought to be empty. In health, during digestion the stomach grasps its contents, so that after a meal an area of dulness rather than of resonance should become apparent. Even in acute indigestion the stomach grips its contents, a grip which often causes "stomach ache." But in atony, with or without static dilatation, an area of resonance at the cardiac end will be found almost immediately after a meal, and even four or five hours later the vault remains high and behaves capriciously under gaseous influences; at no time does it contract in any regular periods. Thus the pyloric portion, unable to withstand the gravitation of its contents, increases its comparatively small transverse diameter, until the stomach may assume a uniform, thick, sausage shape, the upper outline of which may include the nipple and the lower the navel. Dilated colon may be mistaken for a dilated stomach, and in rare cases the mistake may be unavoidable, but in the vast majority of instances by discriminating percussion, even without intubation, these two organs may be distinguished. In difficult cases syphonage or insufflation may help us.

Professor Allbutt says that in his experience the tuning-fork, auscultopercussion, and coin-tapping add little or nothing to what careful percussion can reveal. In a healthy stomach no "squelchy" sounds should be obtainable. They are usually obtainable in atonic dilatation, but rarely amount to the succussion splash of obstructive dilatation. If before breakfast "squelching" can be obtained after the drinking of a tumbler or two of water, we have to deal with a flabby and distended stomach. It is unfortunate that the use of the tube is so resented among private patients as to make such little way as it does in England, for the indications which it gives are of cardinal importance. The normal fasting stomach contains at least from 20 to 30 cubic centimetres of residual fluid, and may hold even 100, but quantities about 100 cubic centimetres are morbid. Moreover, in health this residuum should not contain any particles recognisable as remnants of food; seeds, grit, or fibre in vomit or stool should be carefully noted and the date at which they were swallowed ascertained. The larger the residuum and the more obvious the relics of food the worse the case. As to treatment, considerable stress was laid upon regulation of the diet, the hours of meals, and general tonic regimen, and particular reference was made to the benefit which sufferers from heart disease sometimes experienced from the careful treatment of an associated condition of dilatation of the stomach.

Sir William Broadbent confirmed the frequency of the occurrence of dilatation and atony of the stomach. As to the symptoms of atonic dilatation they were in his experience largely nervous, and among those symptoms which were not generally recognised, was the occurrence of anginoid pains. This spurious angina was apt to come on at night, or after rest, or after meals, instead of after exercise as in true angina. Palpitation and vertigo also resulted from dilatation, the vertigo being an unsteadiness, or feeling of loss of balance, which was very often relieved by eructation of wind. Attacks of asthma at night, and nocturnal symptoms of other kinds, were in his belief usually due to dilated stomach, and were probably of mechanical origin. As to treatment, he said that he had been in the habit of relying upon lavage of the stomach with sulpho-carbolate solution. This should be done last thing at night, rather than in the morning, for a large proportion of the symptoms arose from decomposition within the stomach during the night.

